# Supplementary material for: Isolation and identification of endophytic fungi from Conyza blinii that exhibit antioxidant and antibacterial activities
Source: PeerJ. 2025 May 20;13:e19464. doi: 10.7717/peerj.19464 (PMC12101443; doi:10.7717/peerj.19464)
Supplement: Supplemental Information 7 [file peerj-13-19464-s007.docx]

Supplementary Table 1 Primers information

| Primers | Primer sequence (5'→3') |
| --- | --- |
| ITS1 | TCCGTAGGTGAACCTGCGG |
| ITS4 | TCCTCCGCTTATTGATATGC |

Supplementary Table 2 PCR reaction system

| Fungal gDNA | 1 µL |
| --- | --- |
| ITS1 | 1 µL |
| ITS4 | 1 µL |
| Max PCR Master Mix(2×Premix) | 12.5 µL |
| ddH_2_O | 9.5 µL |
| Total volume | 25 µL |

Supplementary Table 3 PCR reaction condition

| Phase | Cycle index | Temperature | Time |
| --- | --- | --- | --- |
| Predegeneration | 1× | 95℃ | 5 min |
| Reaction | 30× | 95℃ | 30 s |
|  |  | 60℃ | 40 s |
|  |  | 72℃ | 50 s |
| Supplementary extension | 1× | 72℃ | 10 min |
| Preserve |  | 12℃ | ∞ |

Supplementary Table 4 Endophytic fungi isolated from different organs of *C. blinii*.

| Plant organ | Endopyte fungi number | Total |
| --- | --- | --- |
| Root | CBF1, CBF2, CBF5, CBF7, CBF12, CBF13, CBF15, CBF18 | 8 |
| Stem | CBF3, CBF4, CBF6, CBF9, CBF10, CBF14, CBF17 | 7 |
| Leaf | CBF8, CBF11, CBF16, CBF19, CBF20 | 5 |

Supplementary Table 5 The homologous strain of endophyte fungi from *C. blinii*.

| Endopyte fungi | Most closely related strain | Ident(%) | Accession |
| --- | --- | --- | --- |
| CBF1 | *Epicoccum thailandicum* | 98.98% | NR_152926.1 |
| CBF2 | *Epicoccum viticis* | 99.57% | NR_158267.1 |
| CBF3 | *Pseudohumicola lutea* | 97.85% | NR_189397.1 |
| CBF4 | *Fusarium hechiense* | 98.84% | NR_182846.1 |
| CBF5 | *Fusarium pseudoanthophilum* | 97.82% | NR_163682.1 |
| CBF6 | *Fusarium bactridioides* | 98.18% | NR_120262.1 |
| CBF7 | *Fusarium torreyae* | 93.71% | NR_172378.1 |
| CBF8 | *Fusarium hechiense* | 97.74% | NR_182846.1 |
| CBF9 | *Fusarium foetens* | 99.59% | NR_159865.1 |
| CBF10 | *Fusarium circinatum* | 96.02% | NR_120263.1 |
| CBF11 | *Paracremonium apiculatum* | 99.60% | NR_172839.1 |
| CBF12 | *Epicoccum djirangnandiri* | 99.55% | NR_173179.1 |
| CBF13 | *Trametes ellipsoidea* | 90.66% | NR_171793.1 |
| CBF14 | *Humicola olivacea* | 97.25% | NR_147677.1 |
| CBF15 | *Cosmospora lavitskiae* | 99.40% | NR_182322.1 |
| CBF16 | *Paracremonium ellipsoideum* | 99.41% | NR_172840.1 |
| CBF17 | *Epicoccum catenisporum* | 99.16% | NR_158229.1 |
| CBF18 | *Fusarium panlongense* | 98.46% | NR_182847.1 |
| CBF19 | *Fusarium inflexum* | 98.75% | NR_120262.1 |
| CBF20 | *Fusarium acutatum* | 99.21% | NR_111142.1 |

Supplementary Table 6 Chromatographic conditions.

| Chromatographic conditions |  |
| --- | --- |
| Chromatographic column | Waters ACQUITY UPLC HSS T3 Column 1.8 µm, 2.1 mm * 100 mm |
| Mobile phase A | Water containing 0.05% formic acid |
| Mobile phase B | Acetonitrile containing 0.05% formic acid |
| Column temperature | 40℃ |
| Flow rate | 0.3 mL/min |
| Injection volume | 4 μL |

Supplementary Table 7 Gradient conditions for the mobile phase in the chromatography column.

| Time(min) | A(%) | B(%) |
| --- | --- | --- |
| 0 | 95 | 5 |
| 5 | 95 | 5 |
| 13.5 | 5 | 95 |
| 16 | 95 | 5 |

Supplementary Table 8 Mass spectrometry conditions.

| MS conditions. | ESI+ | ESI- |
| --- | --- | --- |
| Duration (min) | 10 | 10 |
| IonSpray Voltage (V) | 5000 | -4000 |
| Temperature (°C) | 550 | 550 |
| Ion Source Gas1 (psi) | 50 | 50 |
| Ion Source Gas2 (psi) | 60 | 60 |
| Curtain Gas (psi) | 35 | 35 |
| Declustering Potential (V) | 60 | -60 |
| MS1 Collision Energy (V) | 10 | -10 |
| MS2 Collision Energy (V) | 30 | -30 |
| Collision Energy Spread (V) | 15 | 15 |
| MS1 TOF Masses (Da) | 50~1250 | 50~1250 |
| MS2 TOF Masses (Da) | 25~1250 | 25~1250 |





Supplementary Figure 1 The colony morphology of endophytic fungi isolated from *Conyza blini*.


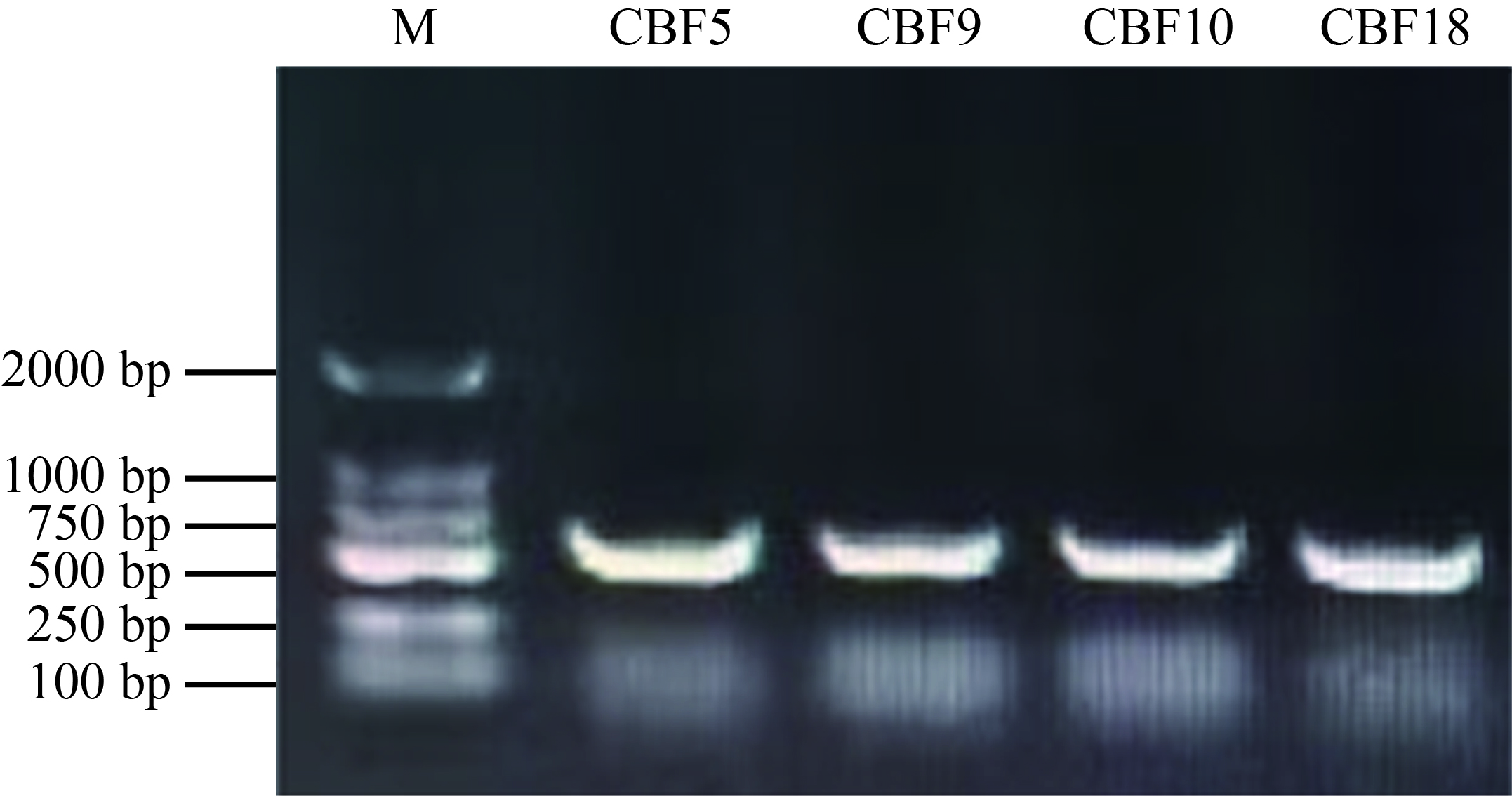


Supplementary Figure 2 Agarose gel electrophoresis of fungal ITS sequences.

（M: Maker D2000）


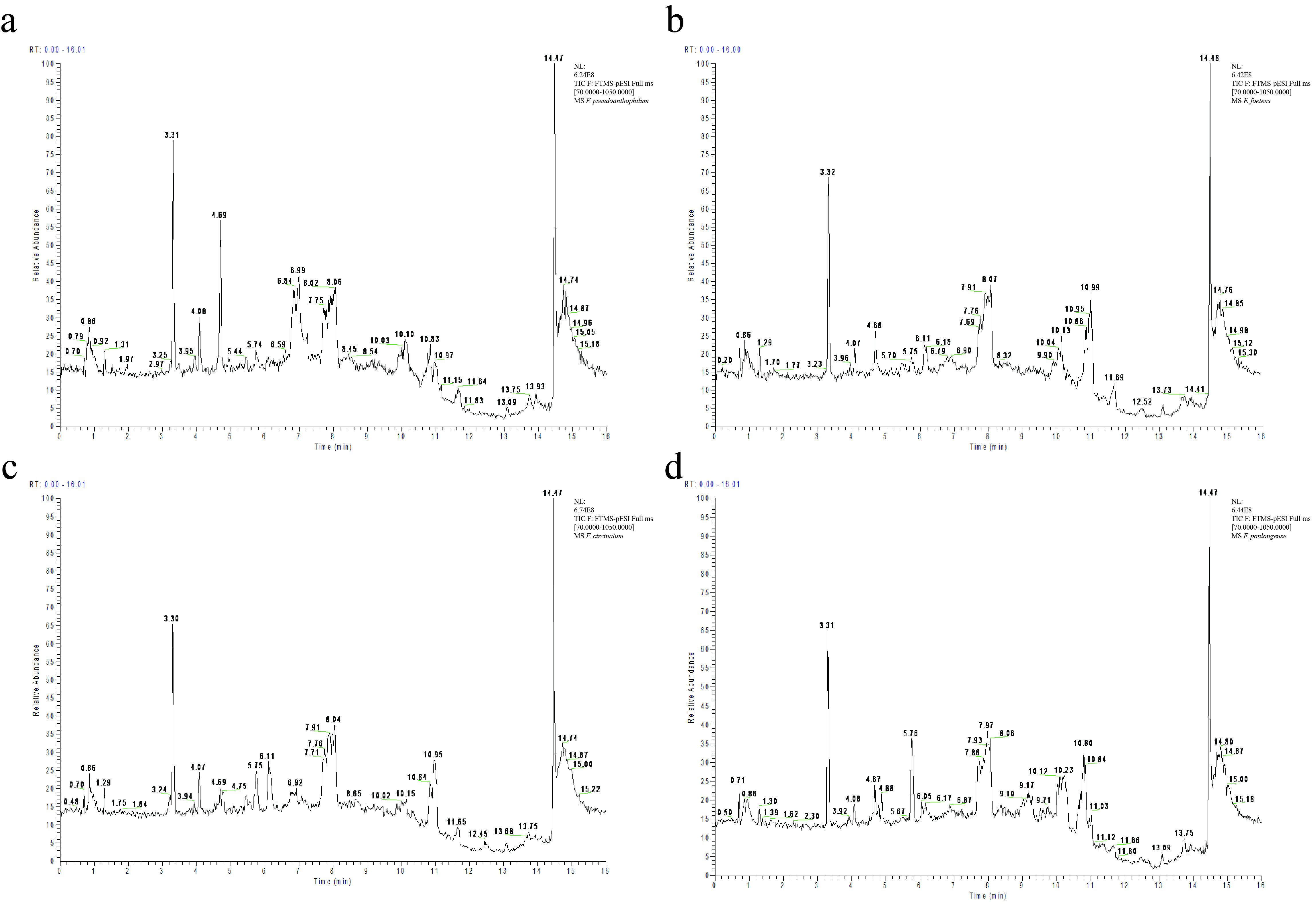


Supplementary Figure 3 LC/MS chromatogram of fungal extracts. a: LC/MS chromatogram of *F. pseudoanthophilum* extracts; b: LC/MS chromatogram of *F. foetens* extracts; c: LC/MS chromatogram of *F. circinatum* extracts. d: LC/MS chromatogram of *F.* *panlongense* extracts.
